# Pharmacodynamics and pharmacokinetics of PLGA-based doxorubicin-loaded implants for tumor therapy

**DOI:** 10.1080/10717544.2022.2032878

**Published:** 2022-02-11

**Authors:** Peng He, Shenglin Xu, Zehao Guo, Peng Yuan, Yulei Liu, Yu Chen, Tiantian Zhang, Yukang Que, Yong Hu

**Affiliations:** a Department of Orthopedics, First Affiliated Hospital of Anhui Medical University, Hefei, China; b Department of Orthopedics, Fuyang Hospital of Anhui Medical University, Fuyang, China; c Department of Pharmacy, Anqing Medical College, Anqing, China; d Laboratory of Pharmaceutical Research, Anhui Zhongren Science and Technology Co., Ltd, Hefei, China

**Keywords:** Doxorubicin, sustained release, intratumoral chemotherapy, PLGA, implants

## Abstract

The traditional systemic chemotherapy through intravenous infusion of doxorubicin (DOX) has many side effects. The aim of this study was to develop a PLGA-based DOX-loaded implant and to evaluate the efficacy and drug metabolism distribution of the implant in intratumoral chemotherapy for osteosarcoma (OS). In this study, implants containing DOX, poly(d,l-lactide-co-glycolide), and polyethylene glycol 4000 were prepared by melt-molding method. Then, the antitumor activity and systemic drug distribution of the implants were tested in a K7M2 OS bearing mouse model. The scanning electron microscope images showed that DOX was uniformly dispersed in the polymer matrix. Both the *in vitro* and *in vivo* release profiles of implants are characterized by three-phase release. Implantation of DOX-loaded implants into tumors can inhibit tumor growth in a dose-dependent manner. The pharmacokinetic behavior shows that intratumor chemotherapy through implants has a much higher drug concentration in tumors than in normal tissues, which may be the reason for improving antitumor activity and reducing systemic side effects. In summary, the drug release of the implants prepared in this study is sustained and stable, which promotes long-term local accumulation of drugs in tumors, improves the efficacy of chemotherapy and has low toxicity to normal tissues.

## 1. Introduction

Osteosarcoma (OS) is a malignant tumor originating from mesenchymal tissues, with an annual incidence of about three cases per million individuals (Bessen et al., 2019). Osteosarcoma is the most common primary malignant bone tumor in adolescents, with approximately 70–80% of patients aged between 10 and 25 years (Lv et al., 2020; Zhao et al., 2021). Osteosarcoma is the second leading cause of cancer-related deaths in children and adolescents (Bagcchi, 2014). Highly malignant and tumor cells are easily transferred to the lungs by blood transport are the two characteristics of OS (Mateu-Sanz et al., 2021). In recent years, with the improvement of neoadjuvant chemotherapy and surgery, more than 90% of patients can receive limb-sparing therapy. The five-year survival rate of patients has also increased to 60–70% (Whelan & Davis, 2018). At present, surgery combined with high-dose, multi-course, and multi-drug sequential chemotherapy is the standard treatment protocol for OS (Jerez et al., 2019; Gill & Gorlick, 2021). Although combined system chemotherapy can improve the limb salvage rate and survival rate of patients, the toxicity of traditional chemotherapy has seriously affected the safety and effectiveness of treatment. The toxicity of systemic chemotherapy is mainly due to the lack of selectivity to tumor tissue, which can damage other normal tissues and cells while killing tumor cells (Li et al., 2012). Also, the lack of drug selectivity will lead to low drug concentration in the tumor area, resulting in poor chemotherapy effects for patients. Increasing the dose of chemotherapy drugs to improve the drug concentration in the tumor can cause additional side effects.

To overcome these obstacles, directly implanting implants with sustained-release drug function into the tumor site through puncture needles or surgery seems to be a promising method for the treatment of tumors (Wang et al., 1999). Implanting biodegradable implants loaded with anti-tumor drugs into the tumor site for local chemotherapy can increase the drug concentration in the tumor, improve the chemotherapy effect, reduce the systemic side effects of chemotherapy, improve drug bioavailability, and improve patient compliance (Al-Abd et al., 2010). Clinically, patients are usually weak after undergoing tumor resection and the surgical wound needs to be healed. Therefore, postoperative chemotherapy can only be carried out after two weeks (Llobat & Gourbault, 2021). However, the tumor cells remaining after surgery are in a period of rapid proliferation at this time. Therefore, intraoperative implants can be placed in the tumor bed to start local chemotherapy as soon as possible, to avoid tumor recurrence caused by residual tumor cells. Carmustine biodegradable implant (GLIADEL^®^ WAFER) is the first local chemotherapy implant approved by the US Food and Drug Administration (USFDA). GLIADEL^®^ has demonstrated excellent efficacy in the treatment of recurrent malignant gliomas (Champeaux & Weller, 2020).

Doxorubicin (DOX) is an anthracycline discovered in the 1960s with anti-tumor effects. It is also one of the most common and effective chemotherapy drugs for OS. Direct insertion of drugs into DNA to affect chromatin, damage topoisomerase-II-mediated DNA repair, and generate free radicals that cause cell damage are the main mechanisms of DOX (Thorn et al., 2011). Although DOX has demonstrated efficacy in OS, common toxic reactions to systemic administration at therapeutic dosages include myelosuppression, alopecia, vomiting, diarrhea, liver damage, and kidney damage, in addition to more serious reactions such as cardiotoxicity (Hood et al., 2016). Cardiomyocyte injury caused by DOX is generally attributed to the generation of oxygen free radicals and oxidative stress that damage the cell membrane, and the degree of cardiomyocyte injury is positively correlated with the dose of DOX (Carvalho et al., 2014). Therefore, the clinical application of DOX is limited. In order to solve these problems, many studies have focused on improving the drug delivery system to enhance the therapeutic effect of DOX and reduce systemic side effects.

To overcome the limitations of DOX in clinical application, we constructed a biodegradable implant containing DOX, poly(d,l-lactide-co-glycolide), and polyethylene glycol 4000 (PEG4000) by the melt-molding method. The implants were characterized in terms of drug content uniformity, micromorphology, drug–excipient compatibility, drug release, and degradability. Then, the antitumor activity of the implants was tested in a K7M2 OS-bearing mouse model. To investigate the pharmacokinetic characteristics of DOX-loaded implants after intratumoral implantation, an accurate and reliable ultra high-performance liquid chromatography–tandem mass spectrometry (UPLC–MS/MS) method was used to detect drug concentrations in tumors, plasma and organs. Finally, the safety of the implant for intratumoral chemotherapy was verified by detecting blood biochemical indicators. The results of this study suggest that continuous intratumoral chemotherapy with DOX-loaded implants can effectively inhibit tumor growth and that increasing the dose of the drug results in higher tumor suppression rates (TSRs) without additional systemic toxicity.

## 2. Materials and methods

### 2.1. Materials

Doxorubicin hydrochloride injection was purchased from Hanhui Pharmaceutical Co., Ltd. (Hangzhou, China). Doxorubicin hydrochloride (Lot: 1050-C180201, purity ≥99.8%) was obtained from Zhejiang Hisun Pharmaceutical Co., Ltd. (Hangzhou, China). PLGA (75:25 lactide/glycolide; intrinsic viscosity 35 mL/g) is produced by Hefei Zhongren Science and Technology Co., Ltd. (Hefei, China). Polyethylene glycol 4000 was purchased from Nanjing Well Pharmaceutical Co., Ltd. (Nanjing, China). For the cell culture, Dulbecco's modified Eagle medium (DMEM) and fetal bovine serum (FBS) were purchased from Biological Industries^®^ (Beit Haemek, Kibbutz, Israel). Glibenclamide was purchased from Sigma-Aldrich (St. Louis, MO). Formic acid was purchased from Tianjin Kermel Chemical Reagent Co., Ltd. (Tianjin, China). Acetonitrile and methanol were purchased from Merck Millipore (Billerica, MA). All of the solvents and reagents for analysis were HPLC grade.

### 2.2. Animals

Specific pathogen-free grade Kunming mice (weighing 28–30 g, male), Sprague-Dawley rats (weighing 200–220 g, male), and BALB/c mice (weighing 13–15.5 g, female, 4–5 weeks) were purchased from Experimental Animal Center of Anhui Medical University (Hefei, China). All animals are kept in a constant temperature (25 ± 2 °C) and 70 ± 5% relative humidity environment. All animal experiments were performed in accordance with the protocol approved by the Institutional Animal Care and Use Committee of Anhui Medical University (Hefei, China).

### 2.3. Preparation of DOX-loaded implants

**Figure 1.**
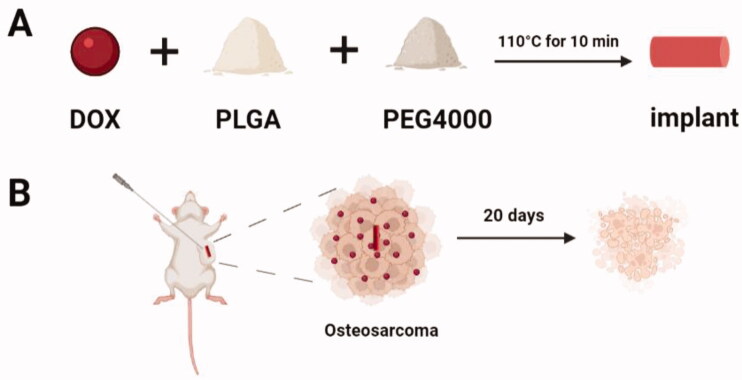
The schematic illustration of the DOX-loaded implant for intratumoral chemotherapy. (A) Preparation of the DOX-loaded implant. (B) A trocar was used to puncture the implant into the tumor center.

The implants containing DOX, PLGA, and PEG4000 (8:15:2 w/w/w) were prepared by the melt-molding method under aseptic conditions (Gao et al., 2019b). Briefly, the dry powders of the three raw materials are purified with a 120-mesh size sieve and then thoroughly mixed. The vacuum-dried mixture is heated at 110 °C for 10–12 minutes until completely melted. The melted mixture was further molded into cylindrical implants for subsequent experiments (Figure 1(A)).

### 2.4. Characterization of DOX-loaded implants

#### 2.4.1. Determination of drug content of the DOX-loaded implants

The drug content in the DOX-loaded implant was determined according to the method described in the Chinese Pharmacopoeia. After the five implants were selected and weighed, they were thoroughly ground with a pestle and mortar. Each ground sample was completely dissolved in 10 mL of mobile phase, and the filtered suspension was centrifuged at 12,000 rpm for 10 minutes. The drug content in 10 μL supernatant was analyzed by high-performance liquid chromatography (HPLC) and the actual drug content of each implant was calculated.

#### 2.4.2. Scanning electron microscopy (SEM)

The surface and cross-sectional morphology of DOX-loaded implants, blank implants, and implants after intratumoral chemotherapy (1, 5, and 20 days) were observed using a Zeiss GeminiSEM300 (Jena, Germany) scanning electron microscope. The images were obtained at 2.5 kV or 5.0 kV accelerating voltage. The samples were coated with gold using a CRESSINGTON 108 automatic sputter coater at 20 mA for 40 seconds before imaging.

#### 2.4.3. In vitro drug release assay

The *in vitro* release characteristics of the implants were detected by the rotating basket method. Fifty milligrams of DOX-loaded implants were placed in a 200 mL release medium containing 0.01 mol/L Tris–HCl buffer (pH = 4.0), and the temperature of the release medium was constant at 37 °C ± 0.5 °C. At predetermined time points (2, 4, 6, 8, 10, 12, 24, 31, and 48 hours), the DOX content in the 5 mL release medium removed from the dissolution apparatus was determined by HPLC. Then, 5 mL of fresh release medium was added back to the dissolution flask.

#### 2.4.4. *In vivo* drug release assay

The implants implanted in the muscles of Kunming mice were taken out at predetermined time points (1, 5, 10, 15, and 20 days) to test the *in vivo* release characteristics of the DOX-loaded implants. The removed implant was rinsed with deionized water and dried, and then the drug content remaining in the implant was measured by HPLC. The *in vivo* cumulative release percentage of DOX was calculated as follows:

$$\text{DOX release percentage }（\%）=\frac{\text{initial DOX amount}-\text{residual DOX amount}}{\text{initial DOX amount}}\times 100\%$$

#### 2.4.5. Differential scanning calorimetry (DSC) analysis

The DSC analysis of the DOX-loaded implant was performed by a thermal analysis instrument Q2000 (TA Instruments, New Castle, DE). First, samples (4–5 mg) of DOX-loaded implants, PLGA, PEG4000, and DOX were sealed in aluminum pans. The parameters of DSC analysis are set to detect in the temperature range of 20–260 °C at a heating rate of 10 °C min^−1^ and use high-purity nitrogen (100 mL/min) as the purge gas.

#### 2.4.6. Fourier-transform infrared spectroscopy (FTIR) analysis

Potassium bromide was added to the DOX-loaded implants, PLGA, PEG4000, and DOX samples, and then they were made into slices by the tableting method. The infrared spectrum of the slices was generated by an FTIR spectrophotometer (Thermo Scientific, Waltham, MA) with a coverage range of 400–4000 cm^−1^. Each generated spectrum was the result of 32 scans with a resolution of 4 cm^−1^.

#### 2.4.7. *In vivo* degradation study

At predetermined time points (7, 14, 21, 28, 35, 42, and 49 days), the blank implants (without DOX) implanted into the muscles of Sprague-Dawley rats were taken out. The removed implant was weighed after being vacuum dried. The percentage of weight loss was calculated according to the formula:

$$\text{Weight loss }（\%）=\frac{\text{initial weight}-\text{residual weight}}{\text{initial weight}}\times 100\%$$

#### 2.4.8. The HPLC method for determination of drug content in the DOX-loaded implants

The HPLC system (Shimadzu, Kyoto, Japan) was equipped with two LC-15C pumps, a SPD-15C essential UV detector, and a CTO-15C essential column oven. A Hypersil BDS C6H5 column (4.6 × 250 mm, 5 μm particle size) was used as the analytical column. The mobile phase consisted of sodium dodecyl sulfate (SDS) solution (1.44 g SDS and 0.68 mL phosphoric acid co-dissolved in 500 mL ultra-pure water), acetonitrile and methanol (500:500:60, v/v/v). The HPLC detection parameters were as follows: the column oven temperature was set at 25 °C; the injection volume was 10 μL; and the ultraviolet (UV) spectrum wavelength was 254 nm. The external standard method was used for quantitative analysis.

### 2.5. Antitumor efficacy of the DOX-loaded implants

#### 2.5.1. Cell culture and K7M2 osteosarcoma bearing mouse model

The K7M2 OS cell line was purchased from Wuhan Procell Life Technology Co., Ltd. (Wuhan, China). The K7M2 OS cells were cultured in DMEM medium containing 10%FBS, 100 U/mL penicillin, and 0.1 mg/mL streptomycin. Before the experiment, procedures such as hair shaving and skin disinfection were performed on the tumor site. 5 × 10^6^ K7M2 cells were resuspended in 100 μL PBS and injected subcutaneously into the right forelimb axilla of each BALB/c mouse (Mochizuki et al., 2021). A palpable tumor appears within 14 days and the *in vivo* study starts when the tumor volume reaches 250–300 mm^3^.

#### 2.5.2. *In vivo* antitumor efficacy

Thirty-six mice were randomly divided into six groups (*n* = 6 per group): blank implant group, DOX solution group (intraperitoneal injections of DOX solution at the dose of 19.5 mg/kg), DOX implants-L group (single intratumoral implantation of low-dose DOX-loaded implants at the dose of 19.5 mg/kg), DOX implants-M group (single intratumoral implantation of medium-dose DOX-loaded implants at the dose of 39 mg/kg), DOX implants-H group (single intratumoral implantation of high-dose DOX-loaded implants at the dose of 78 mg/kg), and DOX implants-Ultrah group (single intratumoral implantation of ultrahigh-dose DOX-loaded implants at the dose of 156 mg/kg). Calculate the dosage of DOX solution in mice according to the usage of DOX (80 mg/m^2^) in clinical chemotherapy for patients with OS (Bispo Júnior & Camargo, 2011). After disinfecting the skin with 75% ethanol, a modified 17-gauge trocar was used to puncture the implant into the tumor center (Figure 1(B) and Figure S1(B)). During the treatment, the tumor volume was measured every two days using a digital vernier caliper. Tumor volume (*V*) was calculated by the formula *V* = 0.5×*L*×*W*^2,^ in which *L* and *W* meant the length and width of the tumor, respectively (Wang et al., 2021). Tumor suppression rate was calculated by the formula TSR=(1 – Wt/Wc)×100%, in which ‘Wt’ and ‘Wc’ meant the final tumor weight of the treated group and blank implant group, respectively. Mice were sacrificed if they had a tumor greater than 20 mm in diameter, or if a tumor became ulcerated or interfered with mobility (Gao et al., 2019a).

### 2.6. *In vivo* safety assessment

During the treatment, the mice in each group were weighed every two days. At the end of the experiment, the mouse blood was collected and allowed to stand at room temperature for two hours. Then, the blood was centrifuged at 3000 rpm for 10 minutes to obtain serum. Serum biochemistry and myocardial enzyme spectrum were detected by Siemens ADVIA 2400 automatic biochemical analyzer (Berlin, Germany).

### 2.7. Histopathological studies

At the end of the experiment, the mice were sacrificed to collect organs (heart, liver, spleen, lung, and kidney) and tumor tissues. The tissue was immersed in 10% neutral paraformaldehyde for 24 hours. The dehydrated tissue was embedded in paraffin and cut into 4 μm thick tissue sheets. Tissue sections were stained with hematoxylin and eosin for histopathological examination.

### 2.8. Pharmacokinetic study

We established a UPLC–MS/MS method to quantitate DOX in plasma and tissues of K7M2 OS-bearing mice. The performance of the UPLC–MS/MS method was fully validated following previous reports, including selectivity, calibration curve, accuracy, precision, recovery, matrix effect, carry-over, dilution integrity, and stability (Gao et al., 2019b).

#### 2.8.1. Pharmacokinetic study in K7M2 osteosarcoma bearing mice

Forty-five K7M2 OS bearing mice were randomly divided into two groups: DOX solution group (intraperitoneal injections of DOX solution at the dose of 19.5 mg/kg, *n* = 20) and DOX implants group (single intratumoral implantation of DOX-loaded implants at the dose of 19.5 mg/kg, *n* = 25). At predetermined time points (6, 12, 24, 48, and 216 hours), the plasma, heart, liver, spleen, lung, kidney, and tumor of the mice were separated and collected to detect the drug concentration in the blood and tissue. Five mice were set at each time point.

#### 2.8.2. Preparation of plasma and tissue samples for UPLC–MS/MS analyses

In brief, 20 μL internal standard (IS) working solution (glibenclamide, 3 ng/mL) was added to 100 μL of plasma and vortex mixed for 30 seconds. Then, 400 μL 0.1% (v/v) formic acid-acetonitrile was added to the above solution and vortex mixed for five minutes. After the mixed liquid was centrifuged at 13,000 rpm for 10 minutes, 5 μL of the supernatant was taken for further analysis.

Mouse tissues (heart, liver, spleen, lung, kidney, and tumor) weighing 30 mg were homogenized in 1 mL ultrapure water containing 0.1% (v/v) formic acid for 15 minutes. After the homogenate was centrifuged at 5000 rpm for 10 minutes, 20 μL IS working solution (glibenclamide, 3 ng/mL) was added to 100 μL of tissue homogenate and vortex mixed for 30 seconds. The subsequent processing procedure was the same as the plasma samples.

#### 2.8.3. Instrument conditions

##### 2.8.3.1. UPLC–MS/MS instrumentations

The LC–MS/MS analysis was performed using an AB Sciex 5500 mass spectrometer coupled with an ExionLC high-performance liquid chromatography system (Framingham, MA).

##### 2.8.3.2. Chromatographic conditions

Column type: Acquity UPLC BEH C18 (Waters Corp., Milford, MA, 50 mm × 2.1 mm, 1.7 μm particles). Mobile phase: the mobile phase was composed of ultrapure water (phase A) and acetonitrile (phase B), both adding 0.1% of formic acid (v/v). Gradient elution program: 0 min, 5% B; 0.5 min, 5% B; 1.2 min, 95% B; 2.2 min, 95% B; 3.0 min, 5% B; 4.0 min, 5% B. Flow rate: 0.2 mL/min. The column oven and autosampler temperature were set to 37 °C and 10 °C, respectively.

##### 2.8.3.3. Mass spectrometry conditions

The optimized parameters for the mass spectrometric analysis were the following: capillary voltage, 2.7 kV; cone voltage, 20 V; desolvation gas flow rate, 650 L/hours; desolvation gas temperature, 350 °C; collision energies, 15 eV. The mass detection was used in the positive electrospray ionization (ESI) mode with multiple reaction monitoring (MRM) for the quantification of DOX and its IS (*m/z* 544.2 → 397.2 and 494.2 → 369.2).

### 2.9. Statistical analysis

Data were statistically analyzed by statistical program SPSS version 22.0 (SPSS Inc., Chicago, IL), and the data were expressed as mean ± standard deviation (SD). The comparison between the multiple groups uses one-way ANOVA, followed by Dunnett’s test to detect intergroup differences. *p< .05 and **p< .01 were considered the difference to be statistically significant.

## 3. Results

### 3.1. Preparation of DOX-loaded implants

The DOX-loaded implants prepared by the melt-molding method were further molded into a cylinder with an average diameter of (0.86 ± 0.03) mm and a length of (4.22 ± 0.26) mm (Figure S1(A)). The average weight of the implants was (3.31 ± 0.19) mg (*n* = 10). Five implants were selected to detect their actual drug content by HPLC. The results showed that the average actual drug content of the implant was (29.78%±0.66%), which was close to the number in the label claim of the drug (32%, w/w) (Table S1) (Figure 1).

### 3.2. Micromorphology of DOX-loaded implants

**Figure 2.**
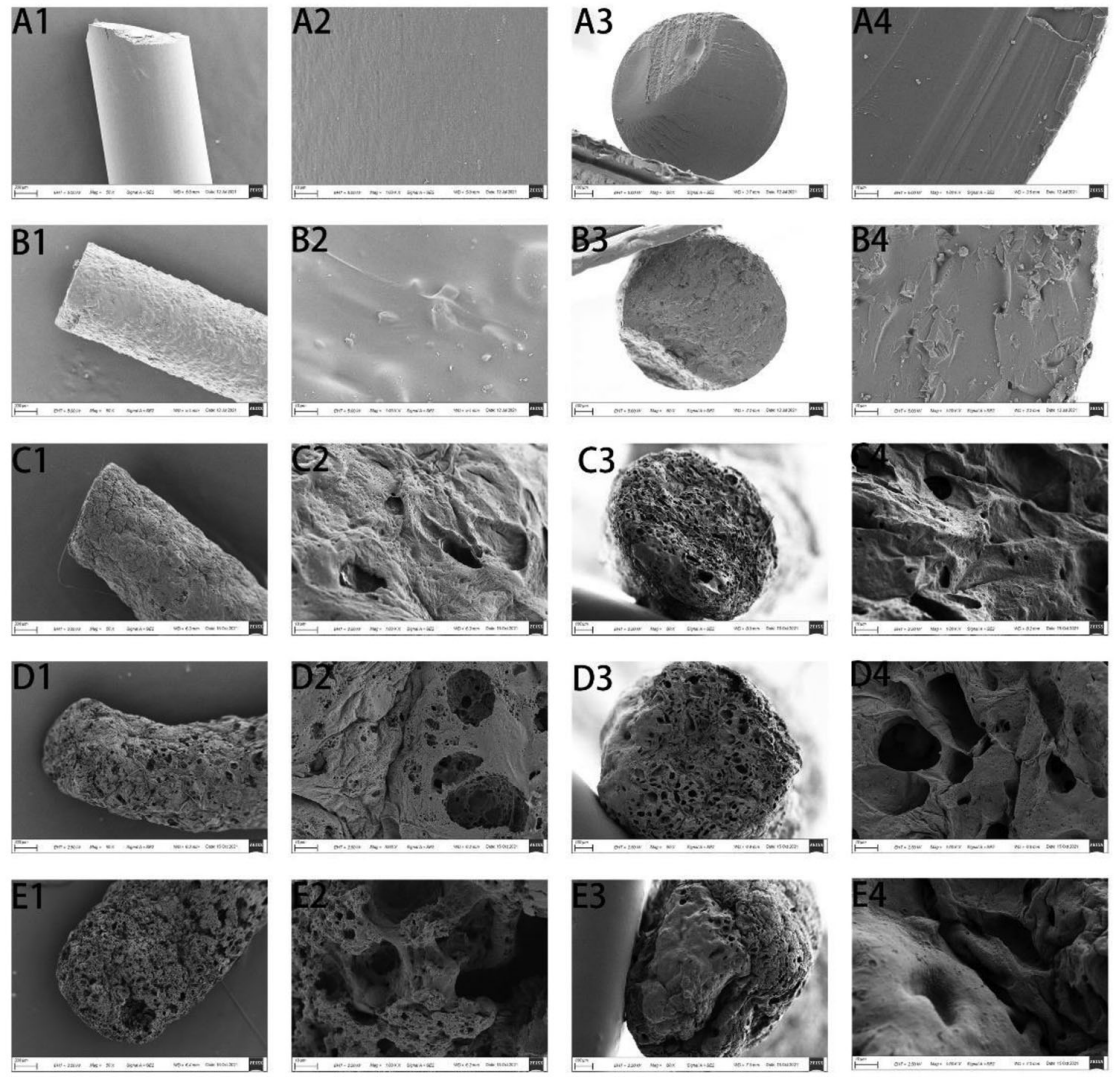
SEM pictures of the DOX-loaded implants. (A1–A4) The blank implant. (B1–B4) The DOX-loaded implant. (C1–C4) The DOX-loaded implant was inserted into the tumor for one day. (D1–D4) The DOX-loaded implant was inserted into the tumor for five days. (E1–E4) The DOX-loaded implant was inserted into the tumor for 20 days. (A1–E1) External surface of the implant (magnification ×50, scale bars: 200 μm). (A2–E2) External surface of the implant (magnification ×1000, scale bars: 10 μm). (A3–E3) Cross-section of the implant (magnification ×80, scale bars: 100 μm). (A4–E4) Cross-section of the implant (magnification ×1000, scale bars: 10 μm).

SEM was used to observe the microstructure and degradation characteristics of the DOX-loaded implants. SEM found that the surface of the DOX-loaded implant was homogeneous without obvious holes and channels. The comparison of the blank implant and the DOX-loaded implant showed that the drug was evenly distributed in the implant (Figure 2(A1–B4)). As the DOX-loaded implant is placed in the body for longer, more and more holes and channels appear on the surface of the implant (Figure 2(C1–E4)). These holes and channels intersect each other inside the implant. On the 20th day, significant erosion of the implant was observed by SEM.

### 3.3. *In vitro* and *in vivo* drug release from the implants

**Figure 3.**
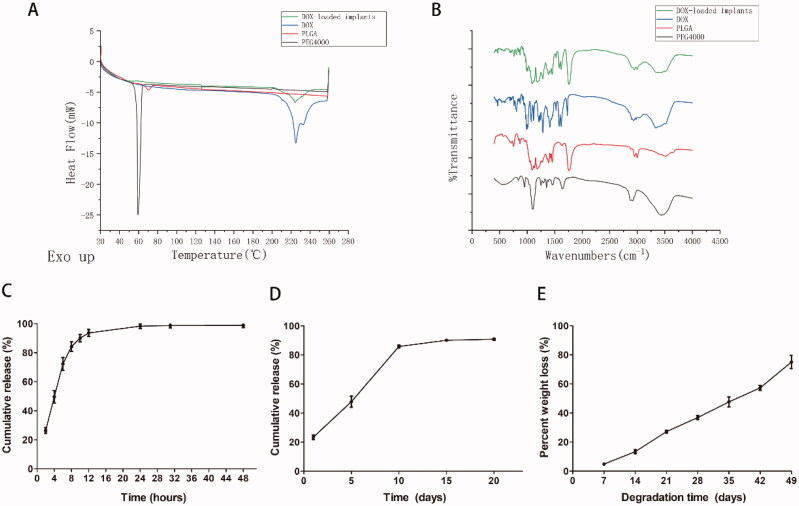
(A) DSC curves of PEG 4000, PLGA, DOX, and DOX-loaded implants. (B) FTIR spectra of PEG 4000, PLGA, DOX, and DOX-loaded implants. (C) The *in vitro* drug release profile of the DOX-loaded implants (*n* = 5 for each time). (D) The *in vivo* drug release profile of the DOX-loaded implants (*n* = 3 for each time). (E) The *in vivo* degradation profile of the blank implants (*n* = 3 for each time).

The *in vitro* and *in vivo* release curves of the DOX-loaded implant were three-phase release, which was characterized by the initial burst release followed by continuous release of DOX, and then enters the plateau phase. The *in vitro* release results showed that the implant released about 26.4% of DOX within two hours (Figure 3(C)). We observed that 84.2% of DOX was released from the implant at a nearly constant rate within eight hours. In addition, the drug was almost completely released within 24 hours.

The *in vivo* release results showed that the implant released about 23.3% of DOX in one day (Figure 3(D)). We observed that 85.8% of DOX was released from the implant at a nearly constant rate within 10 days. In addition, the drug was almost completely released within 15 days.

### 3.4. DSC analysis

The thermal behaviors of DOX, PLGA, PEG 4000, and DOX-loaded implants were analyzed by DSC. The DSC analysis curve of pure DOX showed a broad endothermic peak between 210 °C and 240 °C (Figure 3(A)). The DSC analysis curve shows that the melting sharp endothermic peaks of PEG4000 and PLGA are located at about 60 °C and 70 °C, respectively. In addition, the DSC curves for DOX-loaded implants showed a thermal behavior similar to that of pure DOX and polymers.

### 3.5. FTIR analysis

FTIR spectra analysis of DOX, PLGA, PEG4000, and DOX-loaded implants revealed characteristic absorption bands at different frequencies (Figure 3(B)). From the FTIR spectrum of pure DOX, we observed the characteristic bands at 3552 cm^−1^, 3328 cm^−1^, 2944 cm^−1^, and 1754 cm^−1^, respectively. The typical infrared absorption bands of PLGA and PEG 4000 can be observed in the spectrum of the DOX-loaded implants. In addition, we did not find a new absorption band in the FTIR spectrum of the DOX-loaded implants.

### 3.6. *In vivo* degradation study

The *in vivo* degradation characteristics of the implants were explored by measuring the weight loss of the blank implants at different time points. The experimental results show that the blank implant degrades almost at a constant rate *in vivo* (Figure 3(E)). In addition, the blank implant lost 75% of its original weight on the 49th day. It is difficult to retrieve the remaining blank implants on the 56th day after implantation.

### 3.7. Antitumor efficacy of DOX-loaded implants

**Figure 4.**
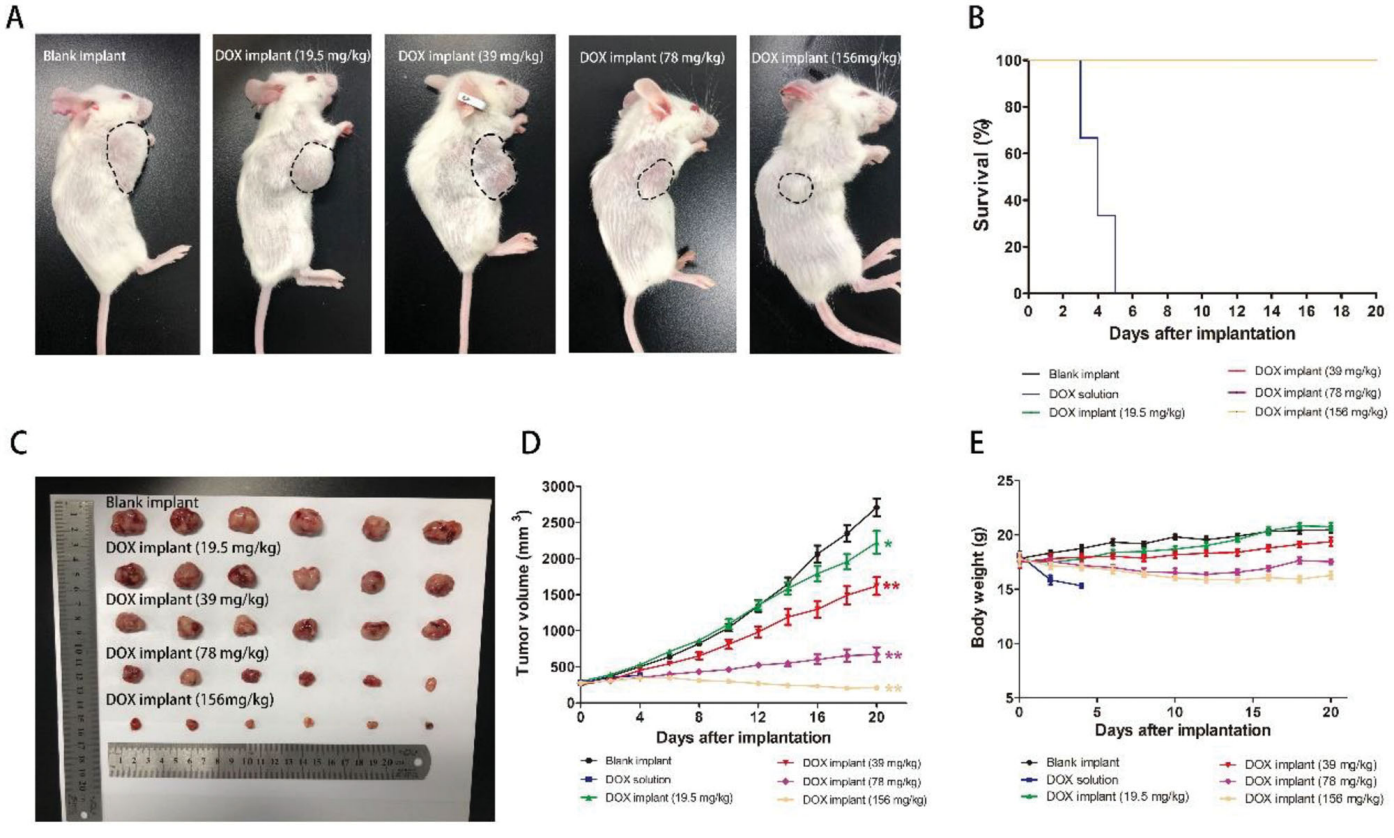
(A) Picture of K7M2 osteosarcoma bearing mice on the 20th day after implantation. (B) Survival curve of K7M2 osteosarcoma bearing mice. (C) Picture of tumors dissected from mice on the 20th day after implantation. (D) Tumor growth curve of K7M2 osteosarcoma bearing mice during the treatment period. (E) The average body weight of mice during the treatment period.

The anti-tumor activity of the implant was evaluated in K7M2 OS bearing mice. Tumors in the blank implant group grew rapidly, which tumor volume exceeded 2700 mm^3^ on the 20th day after implantation. Intratumoral implantation of DOX-loaded implants can inhibit tumor growth in a dose-dependent manner (Figure 4(C,D)). When ultrahigh-dose DOX-loaded implants were given, which was eight times the intraperitoneal injection dose, we observed a significant reduction in tumor volume compared to the other groups.

The mean final tumor weight of the blank implant group was 2.17 ± 0.35 g. The mean final tumor weights were 0.50 ± 0.25 g and 0.13 ± 0.06 g for DOX implant-H and DOX implant-Ultrah groups, respectively (Table 1). The value of TSR in all treated groups exceeded 21%, and the TSR value of the DOX implant-Ultrah group increased to 94%.

**Table 1.** The TSR of blank implants and DOX-loaded implants treated groups.

Groups	Mean tumor weight (g)	TSR (%)
Blank implants	2.17 ± 0.35	
DOX implants-L	1.73 ± 0.38	21
DOX implants-M	1.37 ± 0.31**	37
DOX implants-H	0.50 ± 0.25**	77
DOX implants-Ultrah	0.13 ± 0.06**	94

*p< .05 and **p< .01 were considered the difference to be statistically significant.

**Figure 5.**
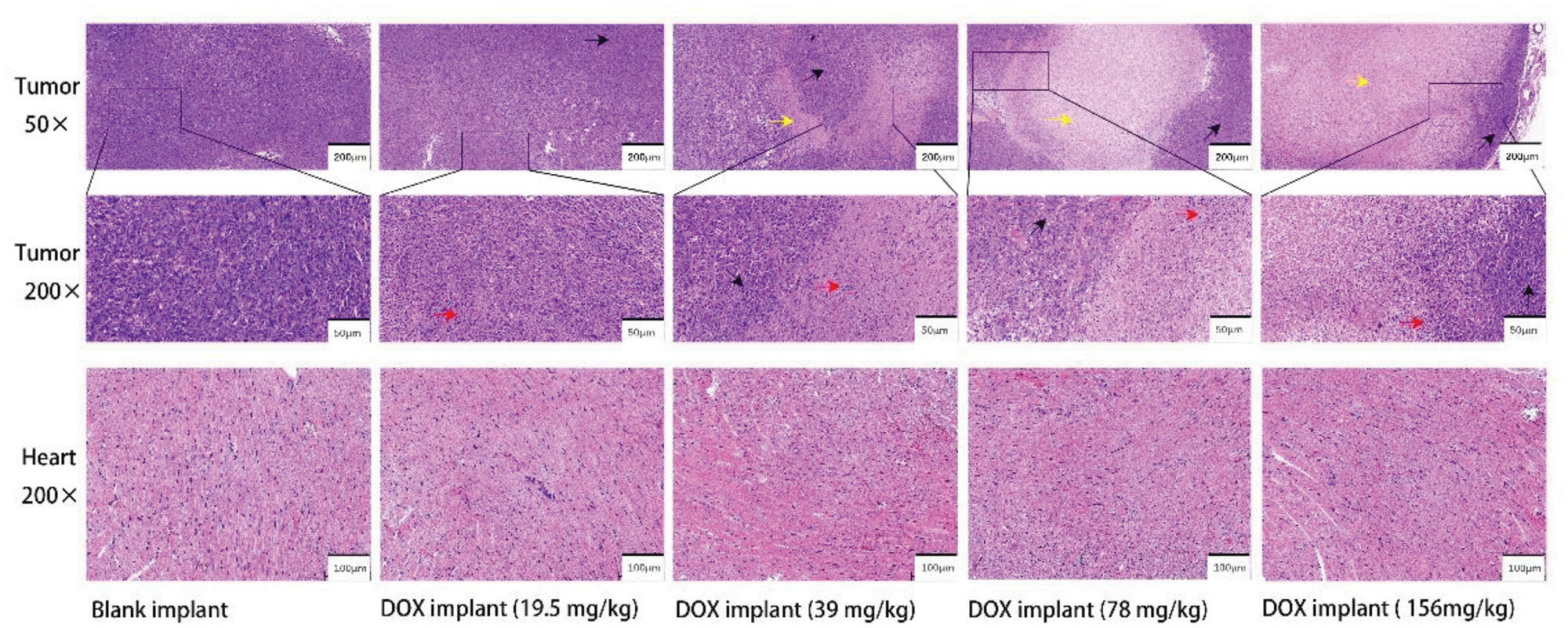
Typical histopathological images of tumor and heart of K7M2 osteosarcoma bearing mice on the 20th day after implantation (yellow arrow represents necrotic area, red arrow represents nuclear debris of tumor cells, and black arrow represents viable tumor cells).

Tumor sections from the blank implant group were composed of close-packed tumor cells, whereas apoptotic tumor cells were rarely observed (Figure 5). However, after treatment by DOX implant-L, typical features including the interstitial space widen, the shrinkage of nuclear and the relative abundance of cytoplasm could be observed. The tumor sections from DOX-loaded implants-(M, H, and Ultrah) treated groups exhibited large necrotic areas mixed with cellular debris.

### 3.8. The safety evaluation of DOX-loaded implant

The mice in each group survived until the end of the experiment except for the DOX solution group (Figure 4(B)). All mice died within five days after receiving the intraperitoneal injection of DOX solution (19.5 mg/kg). During the experiment period, the body weights of mice in blank implant group and DOX implants-(L and M) group increased slowly. However, the body weight of the DOX implants-H group also began to slowly increase after reaching the lowest point on the 12th day of implantation (Figure 4(E)).

**Figure 6.**
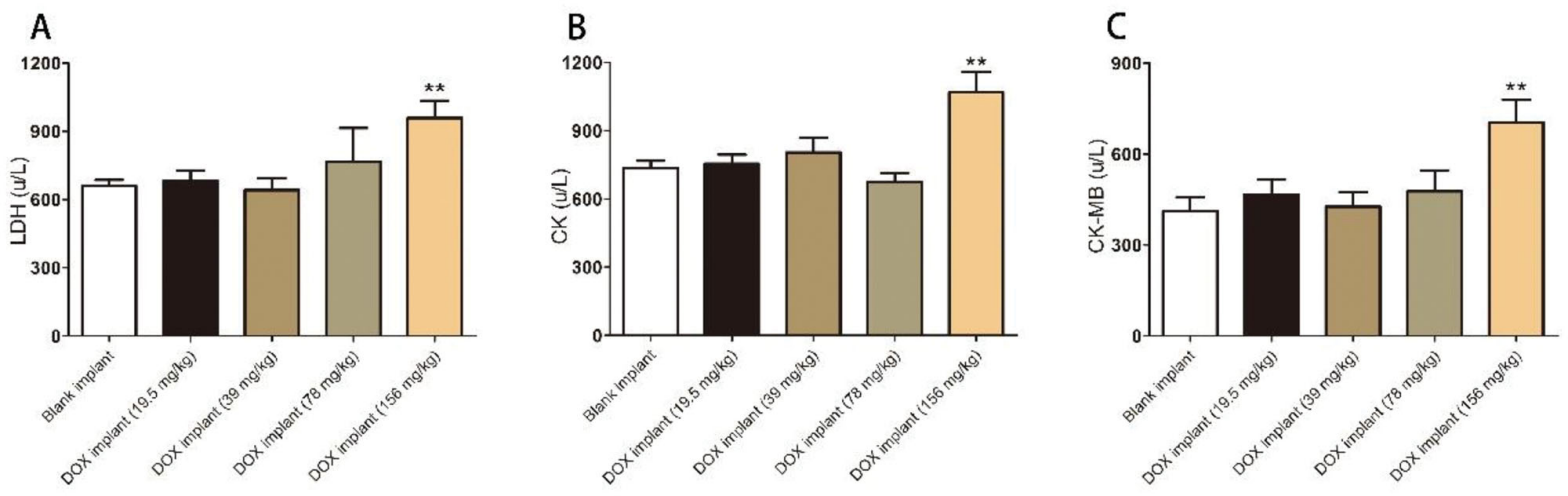
Lactate dehydrogenase (LDH), creatine kinase (CK), and creatine kinase isoenzymes (CK-MB) values of tumor-bearing mice after intratumoral implantation of the DOX-loaded implant for 20 days.

Compared with the control group, the myocardial enzyme detection results showed that only the DOX-loaded implants (156 mg/kg) caused the increase of LDH, CK, and CK-MB in mice (Figure 6). Furthermore, the serum biochemical examination showed that there was no statistically significant difference between the DOX-loaded implants treated groups and the control group (Figure S3).

To further evaluate the systemic toxicity of DOX-loaded implants on the mice, histopathological studies were performed on the major organs of K7M2 OS bearing mice. Based on histopathological observation (Figure 5), a slight disturbance of myocardial fiber arrangement can be observed in the DOX-loaded implants (156 mg/kg) treatment group. Furthermore, we did not find obvious necrosis in the major organs from the DOX-loaded implants treated group (Figure S2).

### 3.9. The pharmacokinetic study and biodistribution of DOX-loaded implant in K7M2 osteosarcoma bearing mice

The DOX concentration was measured by UPLC–MS/MS using the IS method. By measuring the standards of DOX and IS, the retention times of DOX and IS were determined to be 2.58 minutes and 3.90 minutes, respectively (Figure S4(C,D)). No peaks were observed at the elution area of the target analyte in the blank plasma and tissue samples (Figures S4–S10).

**Figure 7.**
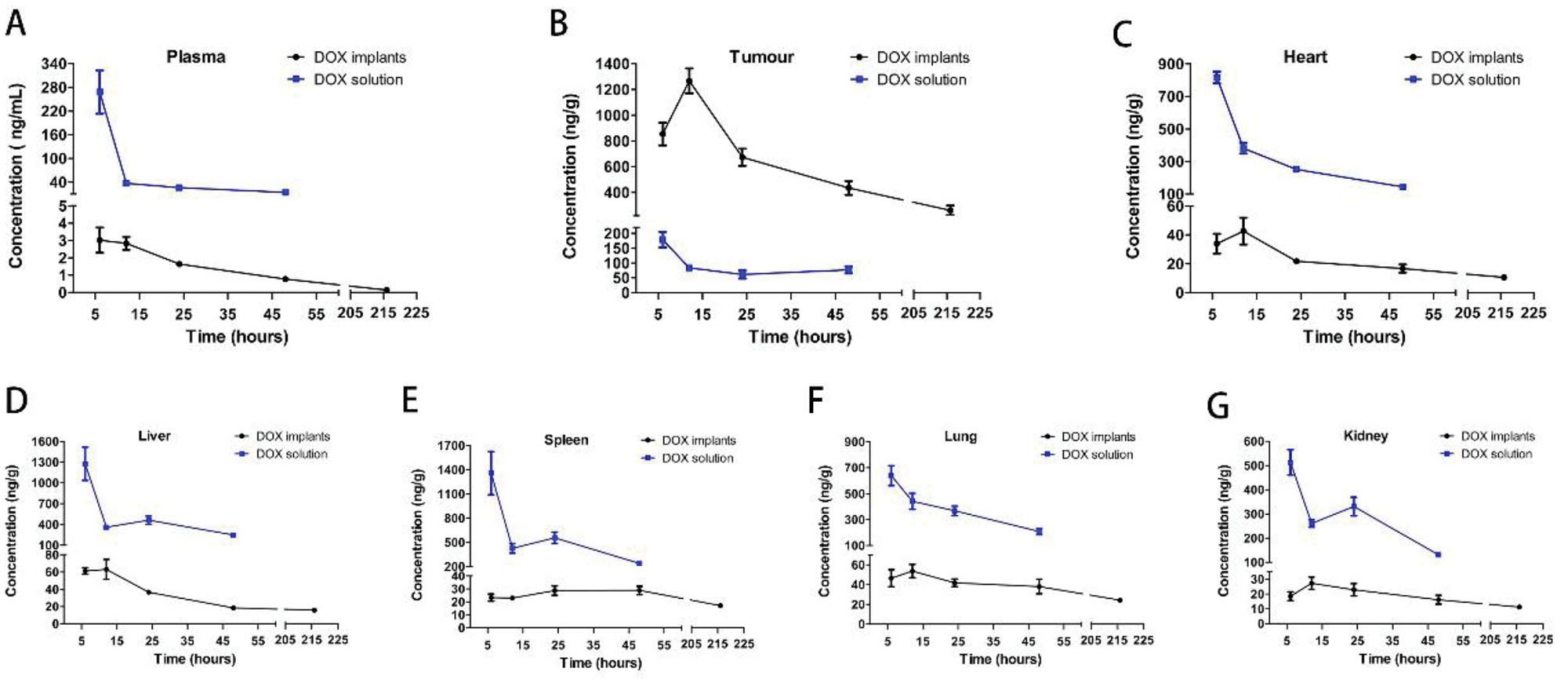
Plasma and tissue concentration–time curves for DOX implants and DOX solution. (A) Drug concentration in plasma at different time intervals. (B) Drug concentration in tumor tissue at different time intervals. (C) Drug concentration in heart tissue at different time intervals. (D) Drug concentration in liver tissue at different time intervals. (E) Drug concentration in spleen tissue at different time intervals. (F) Drug concentration in lung tissue at different time intervals. (G) Drug concentration in kidney tissue at different time intervals.

In the DOX implants group, the DOX concentration in the tumor increased within six hours after implantation, reaching a peak for 12 hours and declining very slow over nine days (Figure 7(B)). Twelve hours after the intratumoral implantation, DOX concentration was the greatest and reached 1266.96 ng/g in the tumor, while DOX concentration in plasma was only 2.83 ng/mL (Figure 7(A)). For comparison, intraperitoneal injection of free DOX at the same dose produced a tumor drug concentration of 178.91 ng/g at the 6th hour, which is significantly lower than the plasma drug concentration of 268.1 ng/mL. Importantly, the DOX tissue levels in the DOX implants group were several orders of magnitude lower compared to systemic delivery at all time points. At the sixth hour after administration, the drug levels in the heart, liver, spleen, lung, and kidney of the DOX solution group were as high as 23.9, 20.8, 58.1, 13.7, and 27.7 times that by the implant group, respectively (Figure 7(C–G)).

## 4. Discussion

One of the most common and useful OS chemotherapy drugs is DOX, which has excellent anti-proliferative and cytotoxic effects on tumor cells (Kazantseva et al., 2021). Traditional chemotherapy through intravenous infusion of DOX has many side effects, such as cardiotoxicity, myelosuppression, vomiting, liver damage, and kidney damage (Hood et al., 2016). The new method of sustained-release delivery of intratumoral drugs has the dual benefits of reducing systemic toxicity while enhancing efficacy for malignant cells. In this study, we developed DOX-loaded implants that directly target the tumor site to prolong drug release, reduce systemic toxicity, and enhance the antitumor efficacy of DOX.

PLGA is an FDA-approved copolymer with excellent biocompatibility and biodegradability, which has been widely used in clinical and medical devices, such as surgical sutures, bone screws, and sustained-release drug delivery systems (Parent et al., 2013; Li et al., 2018). The degradation product of PLGA is lactic acid, which is metabolized by cells and finally degraded into carbon dioxide and water. In addition, the release characteristics of the implant can be adjusted by controlling parameters such as the viscosity, molecular weight, and ratio of PLGA. PEG polymers are characterized by low toxicity, compatibility, and hydrophilicity, which can significantly increase the release of drugs from implants by promoting water diffusion (Wang et al., 2015).

The thermogram of DOX and DOX-loaded implants shows a broad endothermic peak in the range of 210–240 °C, which is related to the melting of DOX (Figure 3(A)) (Gao et al., 2019b). The thermogram of the DOX-loaded implant shows the endothermic peaks of all components and the absence of new endothermic or exothermic peaks, indicating that there is no chemical interaction between DOX and excipients. FTIR analysis can determine whether a drug–excipient reaction occurs from the level of functional groups (Rudra et al., 2010). The presence of DOX in the implant was confirmed by the C–H stretching vibration at 2944 cm^−1^ and the peak at 3328 cm^−1^ (Figure 3(B)) (Neacșu, 2018). The FTIR spectra of the DOX-loaded implant show infrared absorption bands of the functional groups of all components and the absence of new bands, indicating that there is no chemical interaction between DOX and excipients.

The uniform dispersion of the drug in the excipient is the basis for the stable release of the implant (Zhu et al., 2021). The actual drug content of the implant detected by HPLC was (29.78 ± 0.66) %. Low values of SD for the drug content indicate that the drug is evenly distributed in the implant. Furthermore, the SEM image shows that there are evenly distributed small particles on the external surface and cross-section of the implant (Figure 2(B1–B4)). It can be suggested that the manufacturing procedure of the implant yielded the uniform distribution of the drug crystal into the polymeric matrix.

The *in vivo* cumulative release profile of the implants is characterized by three-phase release (Figure 3(D)). This initial burst release is mainly due to the diffusion of the drug accumulated on the surface of the implant. Afterward, almost 60% of the DOX was diffused and released from the implant through the voids left by the released drug and the voids left by the degradation of the excipients. Combining SEM and *in vivo* release curve analysis, it can be concluded that the drug in the implant is released in a diffusion-oriented mode rather than degraded (Figure 2(D1–D4)). The intratumoral implant in our study can rapidly increase the local drug of the tumor to the therapeutic concentration through initial burst release, and then the following sustained-release of the drug could maintain the therapeutic concentration for a long time (Weinberg et al., 2008).

At present, preoperative neoadjuvant chemotherapy combined with surgery is the standard treatment for OS. In the future, DOX-loaded implants can be used as a supplement to these two treatments. First, clinicians can perform debulking of the large tumor before the surgery by puncturing the implant into the tumor (Shikanov et al., 2011). In addition, residual malignant tumor cells and micro-lesions invisible to the naked eye are the main reasons for tumor recurrence. Therefore, placing the implant on the tumor site after resection can effectively kill the surviving tumor cells and prevent tumor recurrence (Yuan et al., 2015). It has been reported that the diffusion rate of the drug in the tumor is inversely proportional to the collagen content in the extracellular matrix and the interstitial fluid pressure (Thiagarajah et al., 2006). It is well known that the tissue pressure in solid tumors is higher than that in normal tissues (Wang et al., 2014; Liu et al., 2019). However, the high pressure in the tumor interstitium significantly prevents intravenous chemotherapy drugs from entering the deep tissues of the tumor (Zong et al., 2015). The results of our study also confirmed that the drug concentration in tissue at six hours after intraperitoneal injection ranked from high to low as follows: spleen > liver > heart > lung > kidney > tumor (Figure 7(B–G)). Therefore, intratumoral chemotherapy through implants can effectively overcome this problem.

Taken together, our experimental data suggest that the therapeutic response of implants is dose-dependent. The 78 mg/kg dose was well tolerated and increased the TSR to 77% (Table 1). Therefore, the excellent therapeutic effect of implants in the treatment of the K7M2 OS-bearing mouse model may be the result of higher doses, slow-release, and prolonged exposure to the drug. Typical apoptotic morphologies including nuclear pyknosis, chromatin agglutination, and nuclear fragmentation can be observed in DOX-loaded implants-(L and M) treated groups. Large necrotic areas mixed with cellular debris can be observed in DOX-loaded implants-(H and Ultrah) treated groups.

All mice died within five days after receiving the intraperitoneal injection of DOX solution (19.5 mg/kg). In this study, DOX was rapidly absorbed into the systemic circulation after intraperitoneal injection, which resulted in the drug being widely distributed in the organs in extremely high concentrations within six hours. The reason for the death of mice may be that the extremely high drug concentration in a short period caused organ damage (Moreno et al., 2010). The DOX-loaded implants increased the maximum tolerated dose of DOX four times compared to systemic delivery, thus potentially improving the antitumor efficacy of DOX in the OS-bearing mouse model. The most serious side effect of DOX is irreversible heart tissue damage (Miao et al., 2012). The myocardial enzyme spectrum results of the DOX-loaded implants-(L, M, and H) treated groups were not statistically different from that of the blank implant group (Figure 6). Pharmacokinetic results confirmed that compared with systemic administration, the cardiac drug concentration in the DOX implant group was several orders of magnitude lower at all time points (Figure 7(C)). It has been confirmed that the degree of cardiomyocyte damage is positively correlated with the dose of DOX (Carvalho et al., 2014). Compared with the blank implant, the serum biochemical indexes of the DOX-loaded implant treated groups are also within the normal range (Figure S3). Moreover, the histopathological examination supported the biosafety of the DOX-loaded implant in major organs (Figure S2). All results demonstrated that the DOX-loaded implants were free of systemic toxicities, offering great promise for clinical applications in OS therapy.

Consistent with the results of the *in vivo* drug release assay of the implant, there was a rapid release of DOX from the implant during the initial burst release phase, and the DOX concentration in the tumor reached a maximum of 1266.96 ng/g at 12 hours (Figure 3(D)). Then, the drug concentration in the tumor was maintained at a relatively stable level from day 1 to day 9 (Figure 7(B)). The favorable sustained drug release and high drug accumulation in tumor cells are also the critical factors of implant for achieving a superior antitumor effect. The results of this study indicate that DOX-loaded implants can inhibit tumor growth in a dose-dependent manner (Figure 4(D)). According to previous studies, we speculate that the unsatisfactory effect of the DOX implants-L group may be due to the insufficient distribution distance of the implant (Gao et al., 2019b). At the sixth hour after administration, the drug levels in the plasma of the DOX solution group were as high as 89 times that of the implant group (Figure 7(A)). Plasma concentrations are often used as a marker of cytotoxic exposure (Shikanov et al., 2011). These results indicated that after loading in the implant the system exposure of DOX remarkably decreased, while more DOX would be accumulated in the site of administration for a long time, leading to low systemic side effects.

Taken together, the DOX-loaded implants have the following advantages: (i) the implant has excellent biodegradability, avoiding the second operation to remove the residual excipient. (ii) The drug and excipients have excellent compatibility. (iii) The implant directly releases the drug inside the tumor can enhance the anti-tumor effect of DOX. (iv) The DOX-loaded implants can improve the biodistribution of the drug and reduce systemic side effects by limiting the accumulation of the drug in normal tissues. (v) The implant can keep the drug at an effective therapeutic concentration for a long time in the tumor.

## 5. Conclusions

In conclusion, we report a biodegradable implant for localized and sustained delivery of DOX to treat OS. The DOX-loaded implants we prepared in this study provided a high local DOX concentration, sustained and stable drug release, extended drug retention inside of tumor, and low toxicity to normal tissues, especially cardiac toxicity. The DOX-loaded implants could significantly inhibit K7M2 OS growth in a dose-dependent manner. Therefore, we conclude that the DOX-loaded implants are a promising drug delivery system in terms of preoperative adjuvant chemotherapy and the prevention of tumor recurrence.

## Supplementary Material

Supplemental Material
