# Supplementary material for: Pharmacodynamics and pharmacokinetics of PLGA-based doxorubicin-loaded implants for tumor therapy
Source: Drug Deliv. 2022 Feb 11;29(1):478–88. doi: 10.1080/10717544.2022.2032878 (PMC8843208; doi:10.1080/10717544.2022.2032878)
Supplement: Supplemental Material [file IDRD_A_2032878_SM7103.docx]

**Table S1.** The drug content of DOX-loaded implants.

| DOX-loaded implants | Label claim of drug  (%) | Actual drug content  (%) | Relative content  (%) |
| --- | --- | --- | --- |
| 1 | 32.00 | 30.66 | 95.81 |
| 2 | 32.00 | 29.09 | 90.91 |
| 3 | 32.00 | 29.38 | 91.81 |
| 4 | 32.00 | 30.27 | 94.59 |
| 5 | 32.00 | 29.50 | 92.19 |


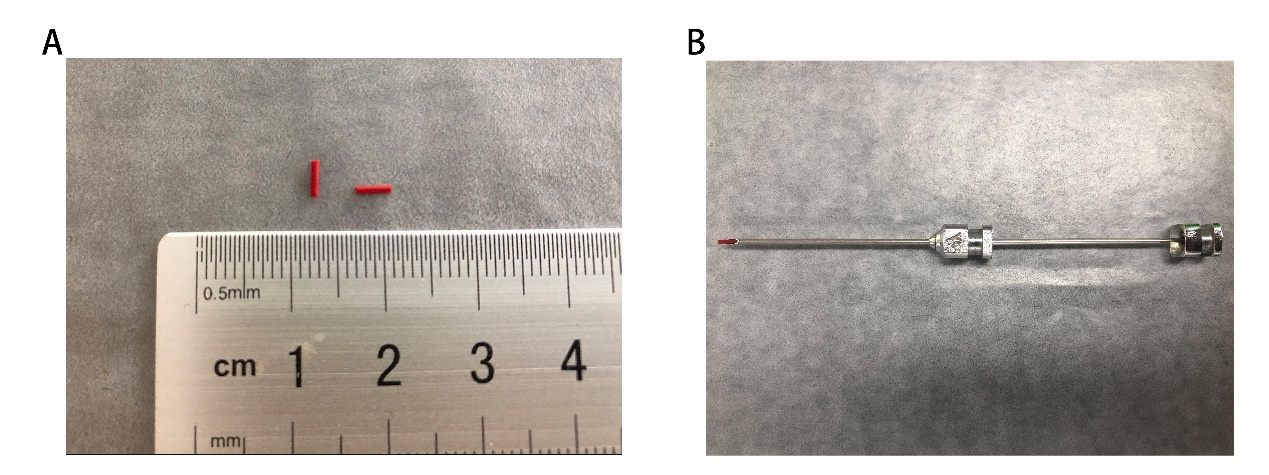


**Figure S1.** (A) Macroscopic picture of DOX-loaded implants. (B) Macroscopic picture of the modified 17-gauge trocar.


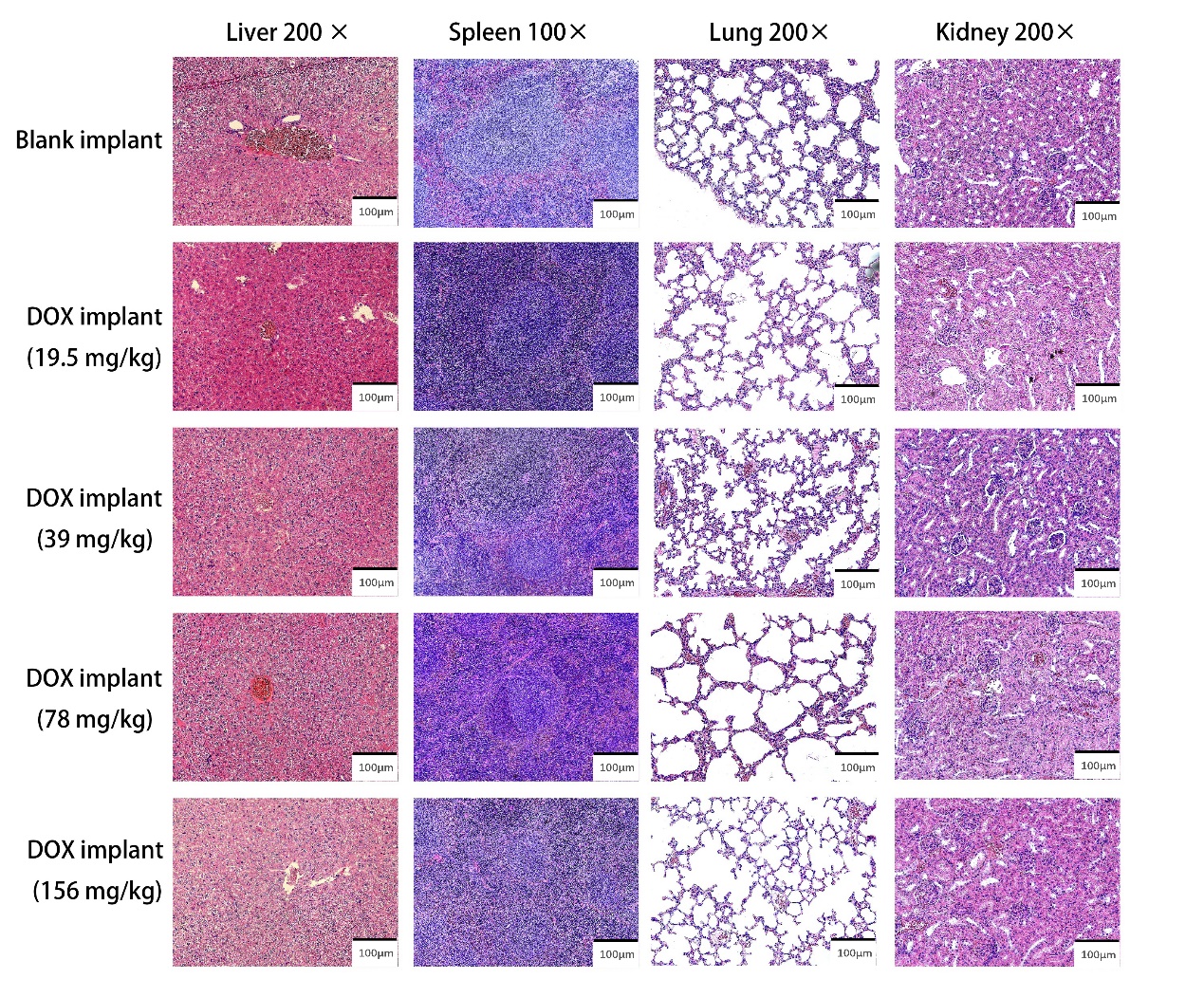


**Figure S2.** Typical histopathological images of major organs of K7M2 osteosarcoma bearing mice on the 20th day after implantation.


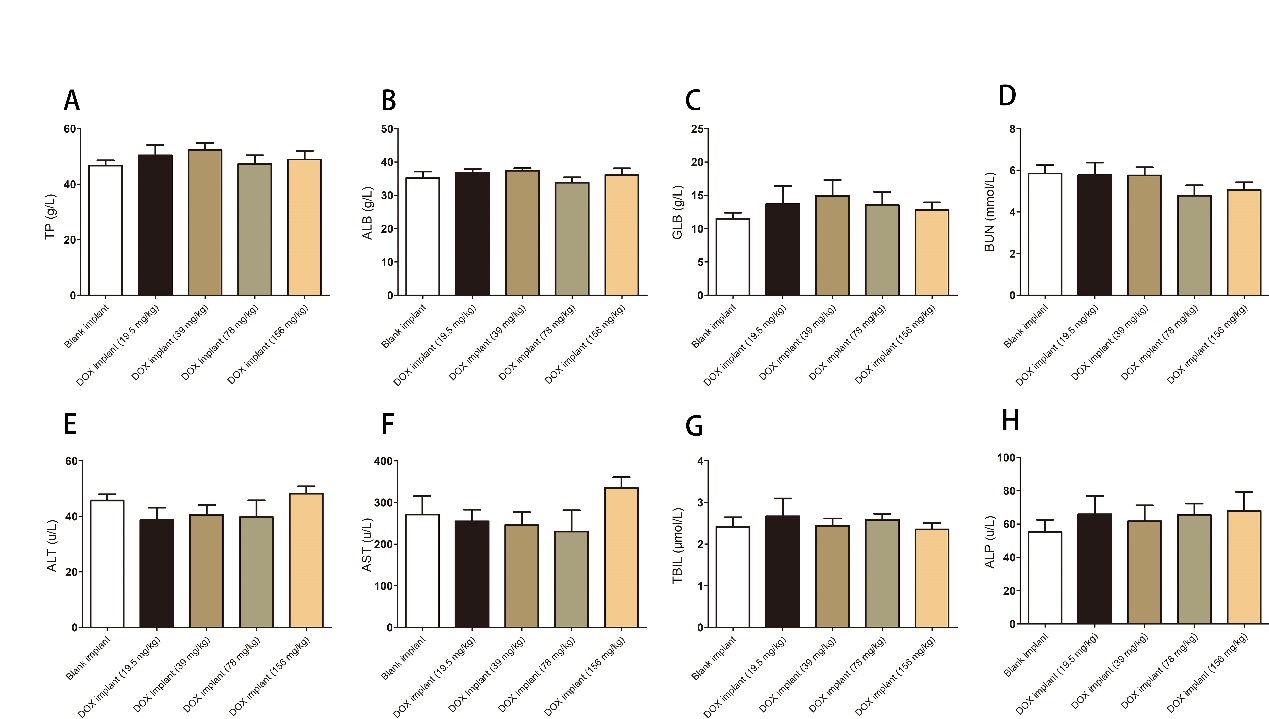


**Figure S3.** Total protein (TP), albumin (ALB) , globulin (GLB) , blood urea nitrogen (BUN) , alanine aminotransferase (ALT) , aspartate aminotransferase (AST) , total bilirubin (TBIL) and alkaline phosphatase (ALP) values of tumor-bearing mice after intratumoral implantation of the DOX-loaded implant for 20 days.


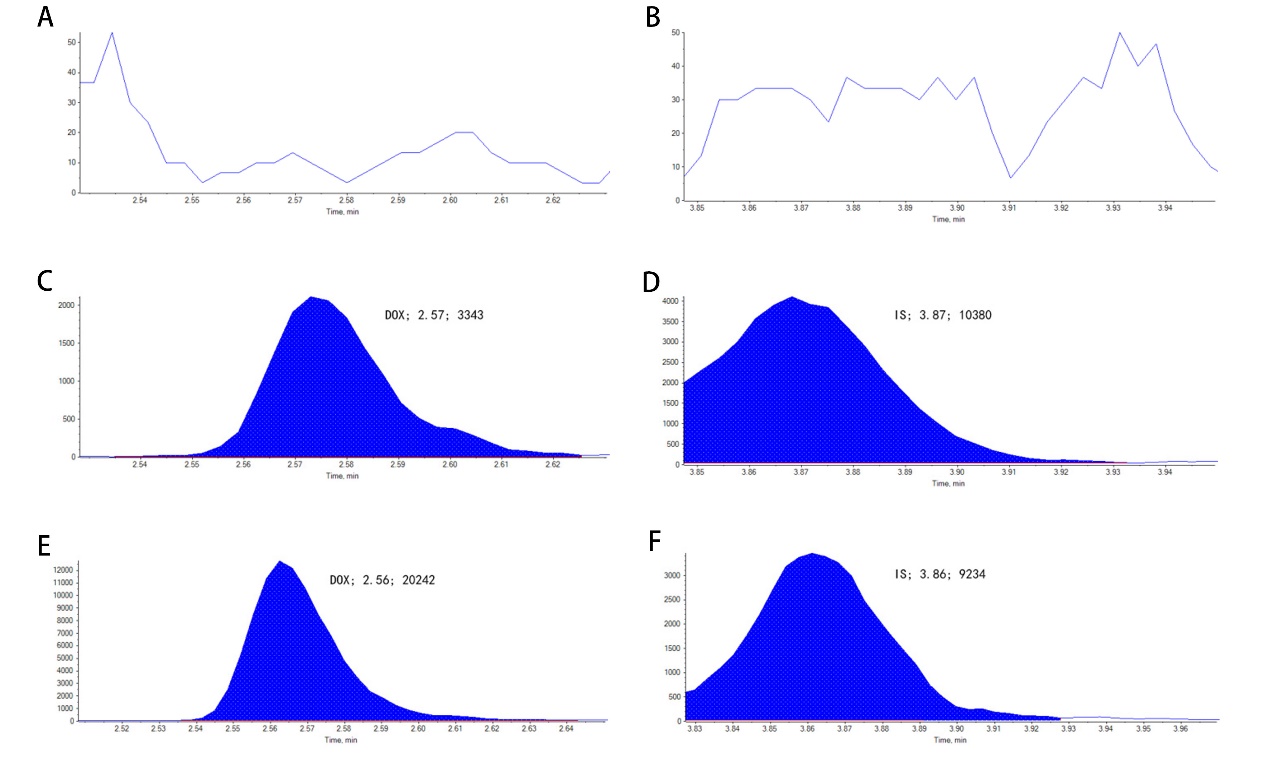


**Figure S4.** Specificity of DOX in plasma samples. (A) The particular chromatograms of blank plasma within the retention time of DOX. (B) The particular chromatograms of blank plasma within the retention time of IS. (C) Blank plasma spiked with DOX. (D) Blank plasma spiked with IS. (E) The chromatograms of plasma within the retention time of DOX. (F) The chromatograms of plasma within the retention time of IS.


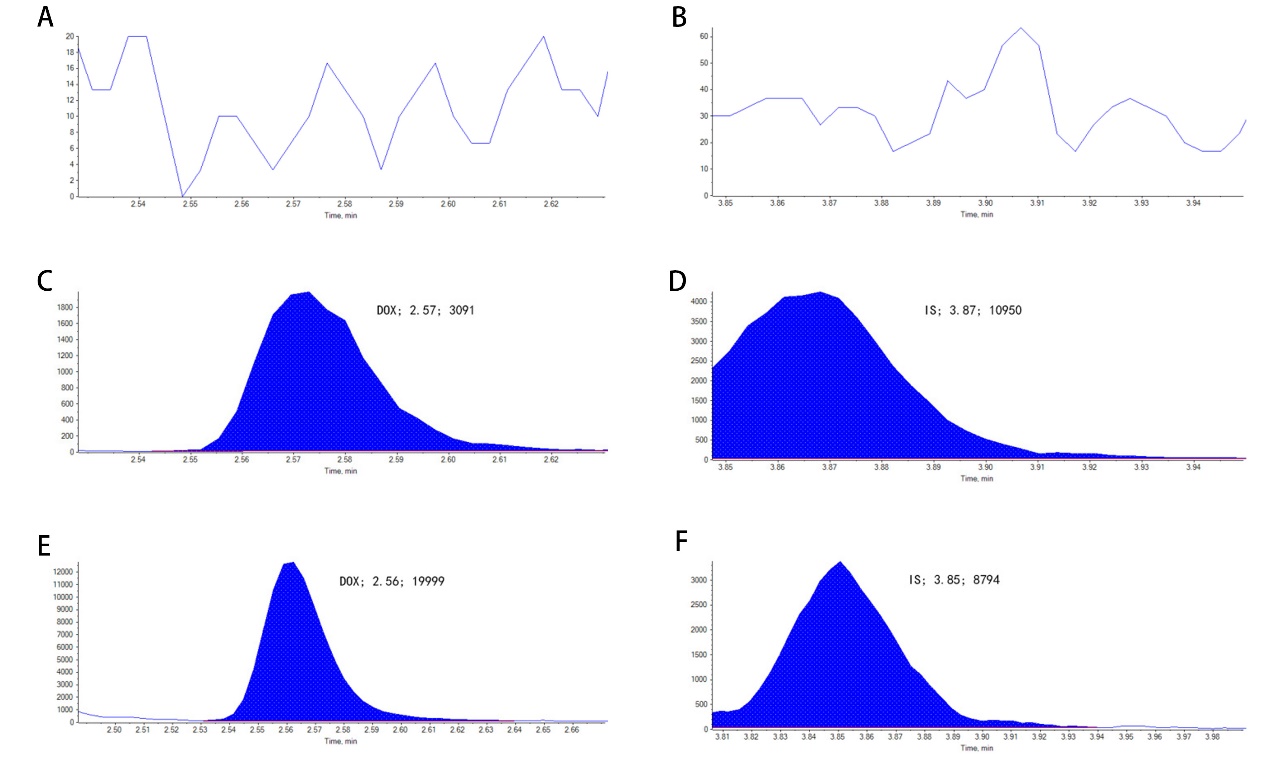


**Figure S5.** Specificity of DOX in tumor samples. (A) The particular chromatograms of blank tumor tissue within the retention time of DOX. (B) The particular chromatograms of blank tumor tissue within the retention time of IS. (C) Blank tumor tissue spiked with DOX. (D) Blank tumor tissue spiked with IS. (E) The chromatograms of tumor tissue within the retention time of DOX. (F) The chromatograms of tumor tissue within the retention time of IS.

**
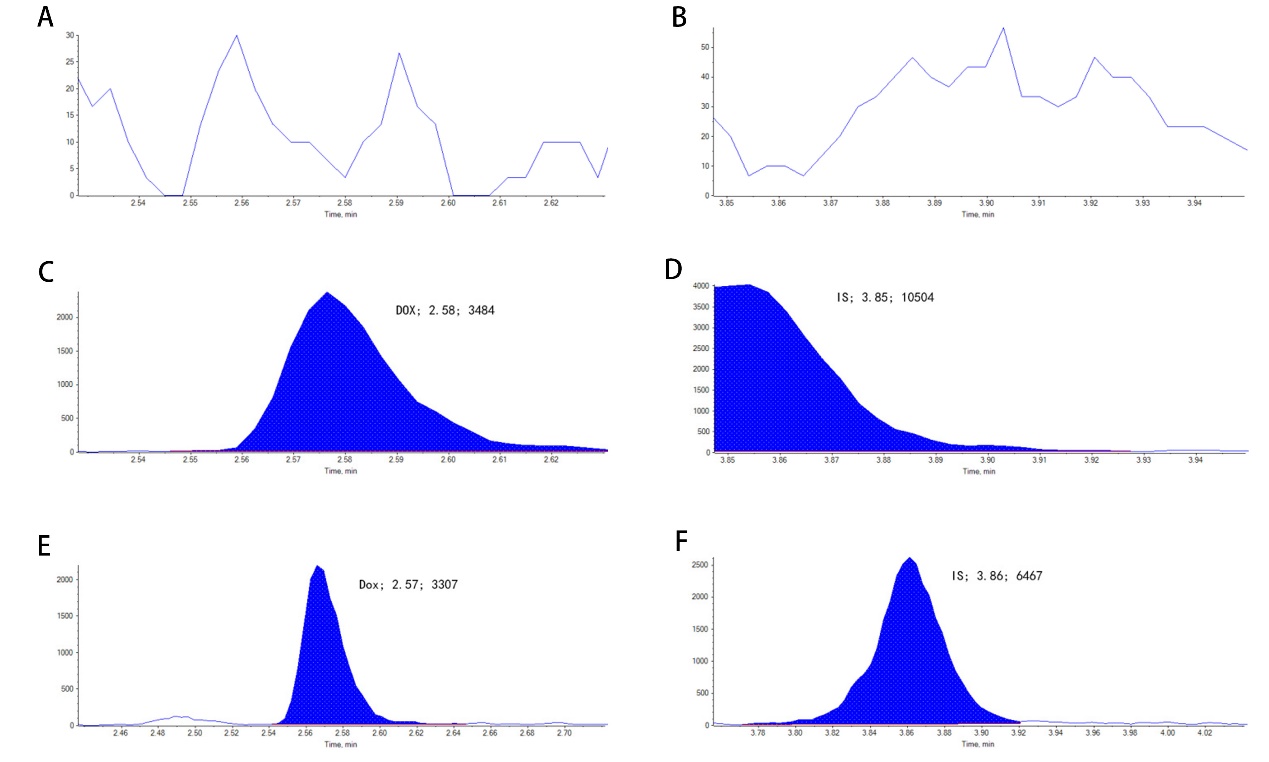
**

**Figure S6.** Specificity of DOX in heart samples. (A) The particular chromatograms of blank heart tissue within the retention time of DOX. (B) The particular chromatograms of blank heart tissue within the retention time of IS. (C) Blank heart tissue spiked with DOX. (D) Blank heart tissue spiked with IS. (E) The chromatograms of heart tissue within the retention time of DOX. (F) The chromatograms of heart tissue within the retention time of IS.


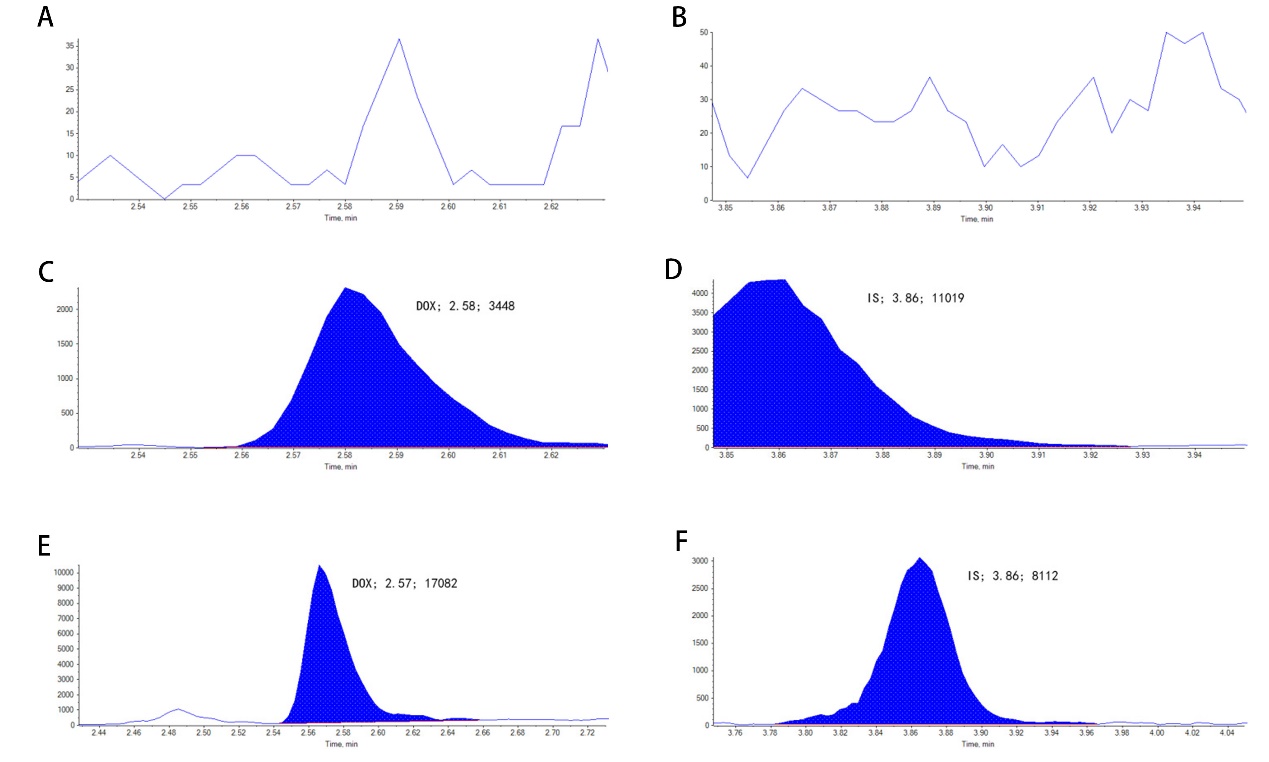


**Figure S7.** Specificity of DOX in liver samples. (A) The particular chromatograms of blank liver tissue within the retention time of DOX. (B) The particular chromatograms of blank liver tissue within the retention time of IS. (C) Blank liver tissue spiked with DOX. (D) Blank liver tissue spiked with IS. (E) The chromatograms of liver tissue within the retention time of DOX. (F) The chromatograms of liver tissue within the retention time of IS.


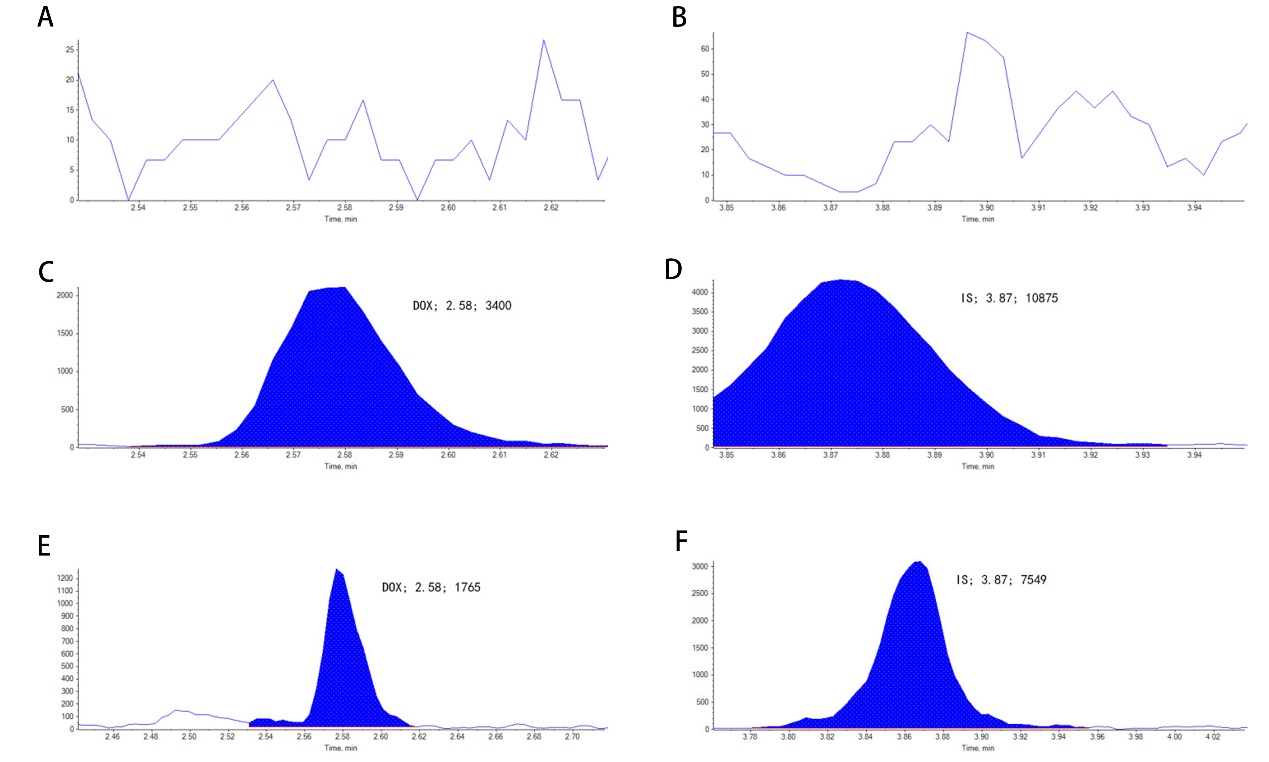


**Figure S8.** Specificity of DOX in spleen samples. (A) The particular chromatograms of blank spleen tissue within the retention time of DOX. (B) The particular chromatograms of blank spleen tissue within the retention time of IS. (C) Blank spleen tissue spiked with DOX. (D) Blank spleen tissue spiked with IS. (E) The chromatograms of spleen tissue within the retention time of DOX. (F) The chromatograms of spleen tissue within the retention time of IS.


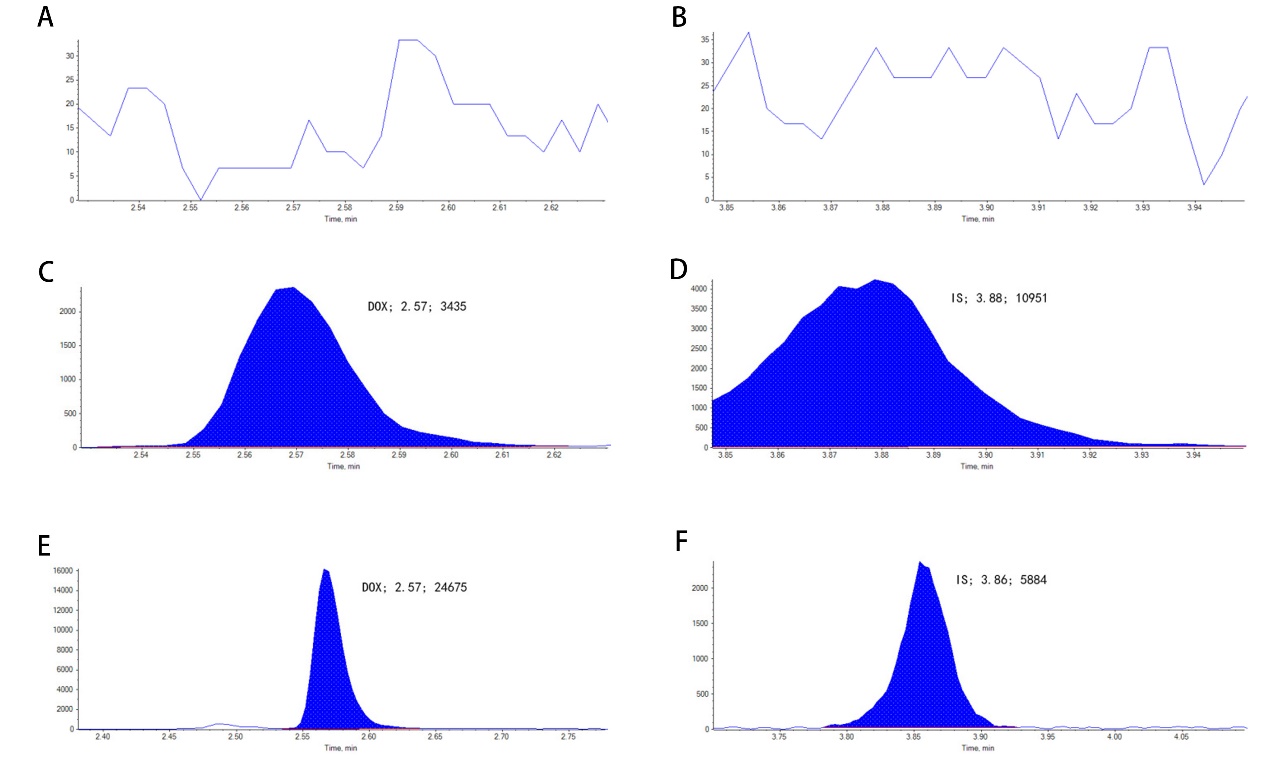


**Figure S9.** Specificity of DOX in lung samples. (A) The particular chromatograms of blank lung tissue within the retention time of DOX. (B) The particular chromatograms of blank lung tissue within the retention time of IS. (C) Blank lung tissue spiked with DOX. (D) Blank lung tissue spiked with IS. (E) The chromatograms of lung tissue within the retention time of DOX. (F) The chromatograms of lung tissue within the retention time of IS.


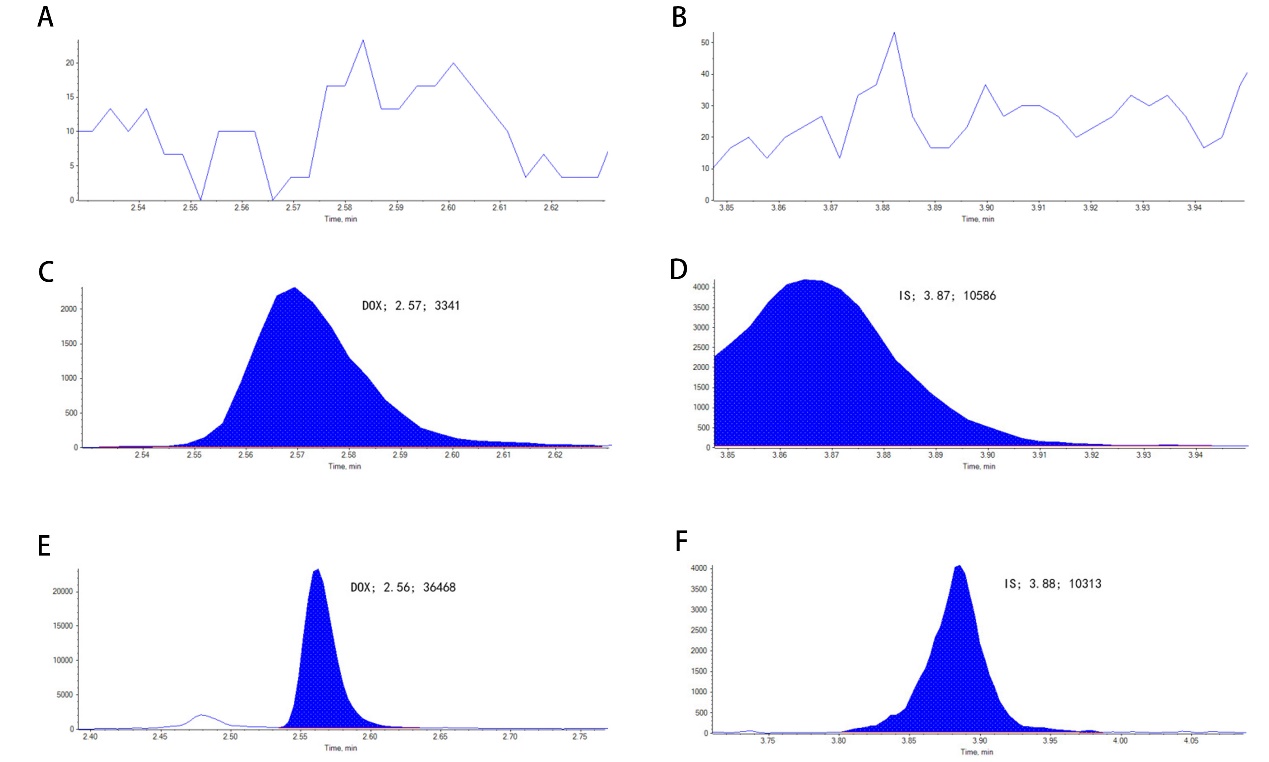


**Figure S10.** Specificity of DOX in kidney samples. (A) The particular chromatograms of blank kidney tissue within the retention time of DOX. (B) The particular chromatograms of blank kidney tissue within the retention time of IS. (C) Blank kidney tissue spiked with DOX. (D) Blank kidney tissue spiked with IS. (E) The chromatograms of kidney tissue within the retention time of DOX. (F) The chromatograms of kidney tissue within the retention time of IS.
